# Transgenerational effect of drug-mediated inhibition of LSD1 on eye pigment expression in *Drosophila*

**DOI:** 10.1186/s12898-020-00330-6

**Published:** 2020-11-23

**Authors:** Sigrid Hoyer-Fender

**Affiliations:** Johann-Friedrich-Blumenbach-Institute of Zoology and Anthropology-Developmental Biology, GZMB, Ernst-Caspari-Haus, Georg-August-Universität Göttingen, Justus-von-Liebig-Weg 11, Göttingen, Germany

**Keywords:** Epigenetic inheritance, LSD1, Tranylcypromine, DMSO, *Drosophila*, PEV

## Abstract

**Background:**

The *Drosophila melanogaster* mutant *white-mottled* is a well-established model for position-effect variegation (PEV). Transposition of the euchromatic *white* gene into the vicinity of the pericentric heterochromatin caused variegated expression of *white* due to heterochromatin spreading. The establishment of the euchromatin-heterochromatin boundary and spreading of silencing is regulated by mutually exclusive histone modifications, i.e. the methylations of histone H3 at lysine 9 and lysine 4. Demethylation of H3K4, catalysed by lysine-specific demethylase LSD1, is required for subsequent methylation of H3K9 to establish heterochromatin. LSD1 is therefore essential for heterochromatin formation and spreading. We asked whether drug-mediated inhibition of LSD affects the expression of *white* and if this induced change can be transmitted to those generations that have never been exposed to the triggering signal, i.e. transgenerational epigenetic inheritance.

**Results:**

We used the lysine-specific demethylase 1 (LSD1)-inhibitor Tranylcypromine to investigate its effect on eye colour expression in consecutive generations by feeding the parental and F1 generations of the *Drosophila melanogaster* mutant *white-mottled*. Quantitative Western blotting revealed that Tranylcypromine inhibits H3K4-demethylation both in vitro in S2 cells as well as in embryos when used as feeding additive. Eye colour expression in male flies was determined by optical measurement of pigment extracts and qRT-PCR of *white* gene expression. Flies raised in the presence of Tranylcypromine and its solvent DMSO showed increased eye pigment expression. Beyond that, eye pigment expression was also affected in consecutive generations including F3, which is the first generation without contact with the inhibitor.

**Conclusions:**

Our results show that feeding of Tranylcypromine and DMSO caused desilencing of *white* in treated flies of generation F1. Consecutive generations, raised on standard food without further supplements, are also affected by the drug-induced alteration of histone modifications. Although eye pigment expression eventually returned to the basal state, the observed long-lasting effect points to a memory capacity of previous epigenomes. Furthermore, our results indicate that food compounds potentially affect chromatin modification and hence gene expression and that the alteration is putatively inherited not only parentally but transgenerationally.

## Background

Epigenetic modifications of chromatin, i.e. DNA methylation and post-translational histone modifications (PTMs), affect chromatin structure and gene activity [1–4]. A predominant mark of pericentromeric heterochromatin in mammals and *Drosophila* is trimethylation (me3) and dimethylation (me2) of lysine 9 of histone H3, respectively, creating a binding site for heterochromatin protein 1 (HP1) [5–7]. H3K9 methylation, therefore, specifies transcriptional repression, whereas H3K4 methylation characterises transcriptionally active regions, as in euchromatin. Both, H3K4- and H3K9-methylation are mutually exclusive [4]. Dynamic regulation of histone PTMs remodels chromatin structure and function. Lysine-specific histone demethylases 1 (LSD1, also known as KDM1A) and 2 (LSD2, also known as KDM1B), encoded in *Drosophila* by the *Suppressor of variegation 3–3* (*Su(var)3–3*), catalyse demethylation of mono- and dimethylated lysines 4 and 9 of histone H3 and are thus able to either repress or activate target genes [8–11]. Due to its dual functions, LSDs have been linked to diverse biological processes including stem and cancer cell biology and have therefore come into focus as potential drug targets [12].

Beyond that, the chromosomal region is important for gene activity as shown by the variegated expression of chromosomally rearranged genes or integrated transgenes, known as position-effect variegation (PEV) [13]. A popular model of PEV is the chromosomal inversion of the X chromosome in *Drosophila melanogaster* causing the relocation of the euchromatic *white* gene to the vicinity of the pericentromeric heterochromatin known as *white-mottled* (In*w*^*m4h*^) [14, 15]. This rearrangement affects the expression of the *white* gene, which encodes a pigment transporter required for the red colour of the eye in wild-type flies. Varying extents of spreading of the condensed and inactive conformation of the heterochromatin over the chromosomal breakpoint into the neighbouring euchromatic gene cause either expression or silencing of *white* in individual cells resulting in a mosaic eye colour phenotype [16–20]. Inactivation is stochastic, but once established, it is stably inherited to daughter cells. Additionally, *trans*-acting genetic modifiers that either enhance or suppress variegation affected silencing [21–23]. The *Drosophila melanogaster* strain comprising the *w*^*m4h*^ rearrangement enabled the isolation of mutations that increase or reduce the number of cells in which silencing occurs, and eventually the identification of enhancers [*E(var)*] and suppressors [*Su(var)*] of variegation. *E(var)*s are mostly transcriptional activators, whereas *Su(var)*s often encode heterochromatin constituents as *Su(var)205*, encoding HP1, *Su(var)3–9*, encoding the H3K9 methyltransferase, and *Su(var)3–3*, encoding dLSD1, which when mutated reduced silencing [24–28].

A heritable phenotype caused by chromatin modification without change in DNA sequence is defined as epigenetic inheritance [29]. Exposure to external factors, such as paternal or maternal diet, or stress conditions may have an inherited epigenetic effect as reported for several species [30–37]. In *Drosophila*, heat stress induces heritable alteration of mottled eye colour mediated by dATF-2 (activation transcription factor 2)-dependent heterochromatin modulation [38, 39]. However, most reports describe in essential parental (or intergenerational) effects, caused for example by in utero exposure of the developing embryo and its germline. In contrast, transgenerational epigenetic inheritance (TEI) is the transmission of a phenotype caused by an induced epigenetic modification to those generations that were never exposed to the signal that triggered the change. TEI would not only be a quick response to environmental changes to broaden phenotypic plasticity but eventually might provide evolvable phenotypic traits [40].

Transgenerational inheritance of epigenetic modification has only rarely been reported [41]. We aimed to interfere with histone PTMs without genetic disturbance of histone modifiers and asked whether the intervention affects gene expression, and in that case, is inherited. As a read-out, we used the well-established *Drosophila* model of PEV *white-mottled,* In*w*^*m4h*^, in which phenotypic changes are easily determined by eye colour expression. Another advantage of using *Drosophila* is that due to its very low level of genome methylation the effect of DNA methylation can be neglected [42]. Flies were fed with trans-2-phenylcyclopropylamine (Tranylcypromine), an irreversible monoamine oxidase inhibitor (MAOI) effectively inhibiting LSD1/2 [43, 44], and its effect on H3K4 methylation and eye colour expression was investigated by quantitative immunoblotting, optical measurement of eye pigment expression, and transcription of *white*. Feeding of Tranylcypromine was additionally chosen to elucidate the relevance of food compounds on histone PTMs and gene expression. Beyond that, the question about a transgenerational effect of epigenetic inhibitors is of crucial importance concerning their wide clinical applications.

Tranylcypromine (trade name Parnate) is clinically used for the treatment of depression, especially when therapy-resistant, anxiety, and Parkinson’s disease [45]. Furthermore, it efficiently inhibits cell proliferation in several cancer cell lines and is thus considered as a potential anti-cancer drug [46]. Medication of Tranylcypromine, an irreversible MAOI, in psychiatric therapy caused an increase of neural concentration of monoamine and indolamine neurotransmitters, including dopamine, epinephrine, norepinephrine, serotonin and tyramine, that are otherwise metabolized and subsequently inactivated by MAOs [47]. Genes, responsible for dopamine synthesis are evolutionarily conserved between mammals and flies. In *Drosophila*, dopamine is required for cuticle synthesis and pigmentation. However, direct orthologs of MAOs, which inactivate dopamine, have not been identified in flies so far indicating that application of Tranylcypromine in flies does not affect neurotransmitter concentration [48].

We found that Tranylcypromine is an effective LSD inhibitor in *Drosophila* thus increasing H3K4 methylation. Feeding of Tranylcypromine and its solvent DMSO not only affected gene expression in treated animals but also showed a long-lasting effect detectable in consecutive generations. An increase in eye colour expression is prominent in treated animals (F1 generation), whereas in the following F2 and F3 generations eye colour expression is strongly silenced. We observed a large variability in eye pigment expression throughout consecutive generations that eventually returned to the original level defined by the control flies. Our results show that drug-mediated desilencing of *white* in F1 affects also consecutive generations that have never been in contact with the additive. Our data exemplify that food compounds are potentially able to alter histone PTMs and gene activity and that these alterations can be inherited transgenerationally.

## Results

### Tranylcypromine inhibits histone H3K4 demethylation in S2 cells

**Fig. 1:**
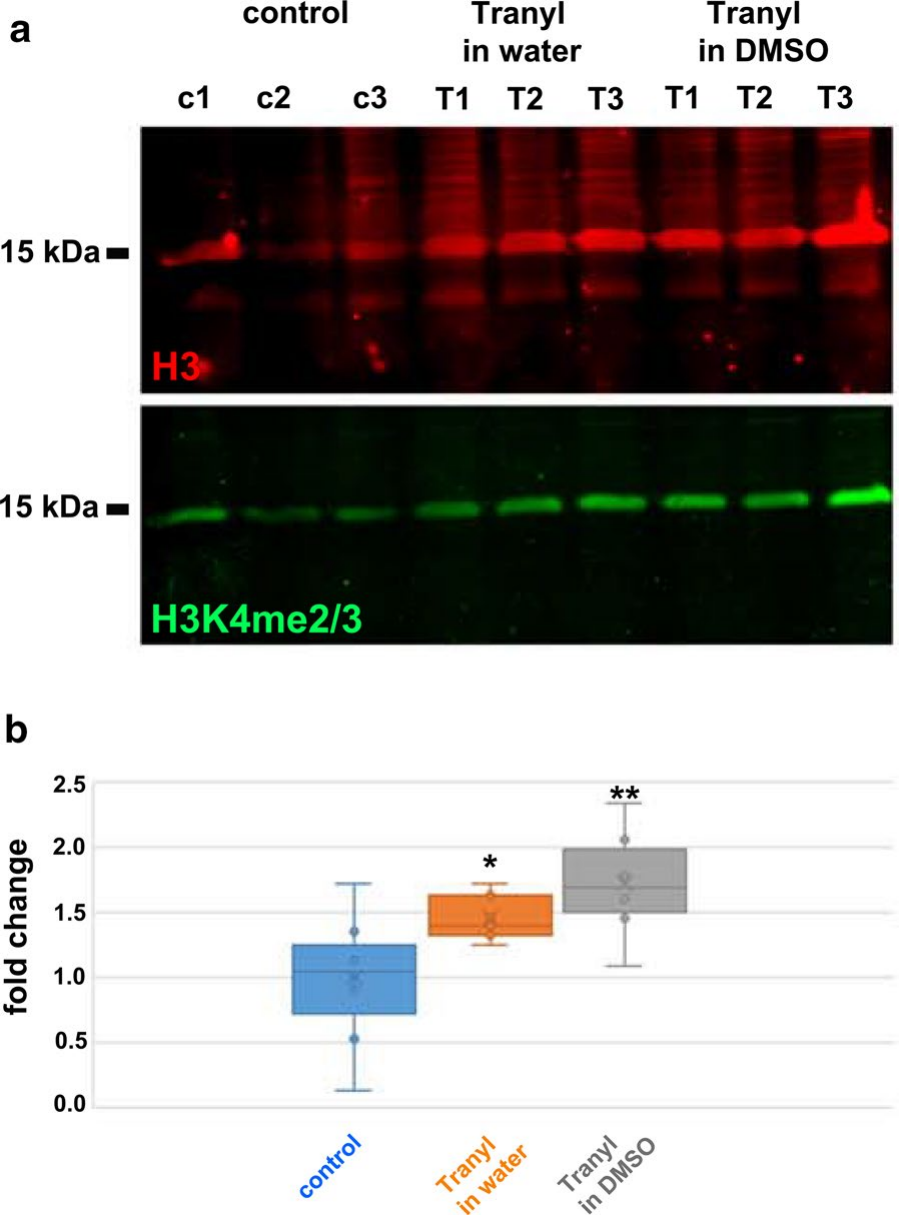
Tranylcypromine treatment of *Drosophila* Schneider’s S2 cells raised methylation of histone H3 at lysine 4 (H3K4). S2 cells were grown in standard medium (control) or in the presence of either Tranylcypromine dissolved in water (Tranyl in water), or Tranylcypromine dissolved in DMSO (Tranyl in DMSO). Cells were lysed and sonified in SDS sample buffer, separated on 15% SDS-PAGE, and histone H3 and di-and trimethylated histone H3K4 detected by fluorescent secondary antibodies. **a** Representative Western blot showing detection of histone H3 (red) and histone H3 di-and trimethylated at lysine 4 (H3K4me2/3; green). **b** Ratio of the quantity of H3K4me2/3 related to H3 and calculated as the fold change of the control. Control (3 biological replicates, n = 9 × loaded), Tranylcypromine in water (3 biological replicates, n = 7 × loaded), Tranylcypromine in DMSO (3 biological replicates, n = 8 × loaded). Data are normally distributed. Significant differences to the control according to Student’s T test (one-tailed, homoscedastic): Tranylcypromine in water p*, Tranylcypromine in DMSO p**

*Drosophila* S2 cells were treated with Tranylcypromine and the amount of di-and trimethylation of H3K4 (H3K4me2/3) related to histone H3 quantified by immunoblotting. Since Tranylcypromine is soluble in both, either water or DMSO, we considered an increased bio-availability of Tranylcypromine when dissolved in DMSO. S2 cells were cultured either in standard medium as control or in the presence of 2 µM Tranylcypromine dissolved either in water or DMSO. Total proteins were immunoblotted, and histone H3 and histone H3K4me2/3 quantified by fluorescent imaging (Fig. 1a). The fold changes of the relative quantities of H3K4me2/3, i.e. the ratios of H3K4me2/3 to H3, related to the mean ratio of the controls on the same blot are given in Fig. 1b. Our results revealed that Tranylcypromine treatment significantly raised the relative amount of H3K4me2/3 (Fig. 1b).[Student’s T test, one-sided, homoscedastic, related to control: Tranylcypromine in water p*, Tranylcypromine in DMSO p**. Three biological replicates each for control and Tranylcypromine treatments. Individual measurements for control n = 9, Tranylcypromine in water n = 7, Tranylcypromine in DMSO n = 8. Data are normally distributed.] The data, furthermore, indicated that DMSO intensifies the effect of Tranylcypromine treatment on H3K4 methylation.

### Enhanced eye pigmentation in generation F1 by feeding with Tranylcypromine

**Fig. 2:**
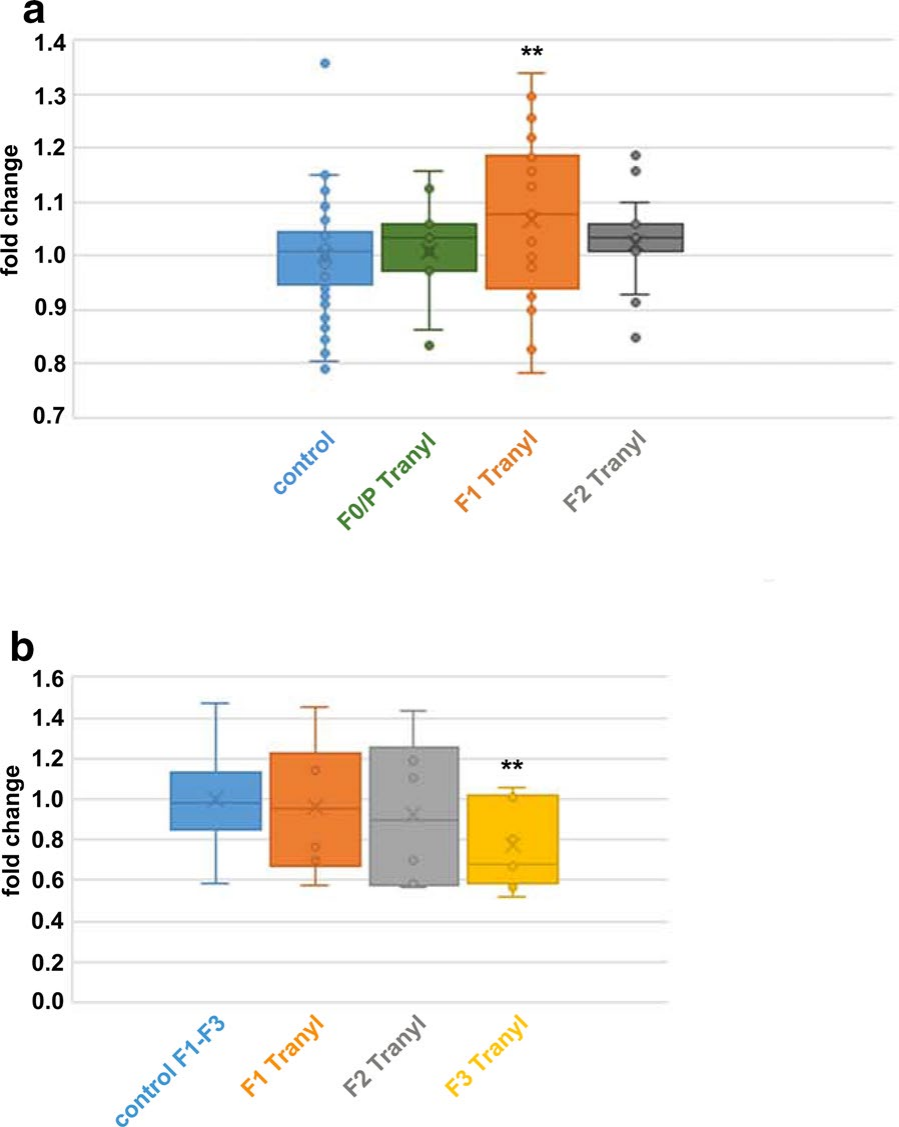
Feeding of Tranylcypromine dissolved in water on eye pigment expression. **a** Eye colour expression in male flies fed with Tranylcypromine (1.25 mg up to 4 mg dissolved in water per culture vessel) in F0/F1 generation. Flies of consecutive generations were flipped onto fresh culture medium without additive, and after egg-laying prepared for analysis. Eye colour measurement was done in triplicates (using 3 × 20 male heads for each probe). Three independent experiments comprising a total of six biological replicates for the control (untreated flies), and 11 biological replicates for Tranylcypromine treatment (control n = 45 × 20 heads, F0/P n = 9x, F1 n = 26x, F2 n = 18x). Student’s T test (one-sided, homoscedastic) to control: F0/P Ø, F1 **, F2 Ø. Data are normally distributed. **b** Relative *white* gene expression in adult male fly heads of consecutive generations after feeding with Tranylcypromine (1.25 mg dissolved in water per culture vessel) in F0/F1 generation. Relative *white* gene expression is significantly different compared to the controls in the F3 generation (p**; Student’s T test, one-sided, homoscedastic). Expression of *white* was related to *actin* expression in each probe and the fold change calculated to the mean relative expression in the control. Control flies (altogether five biological replicates and n technical replicates: F1 (n = 8) + F2 (n = 3) + F3 (n = 8)), F1 flies (one biological replicate and six technical replicates), F2 flies (two biological replicates and six technical replicates), F3 flies (three biological replicates and nine technical replicates)

We asked whether Tranylcypromine, dissolved in water, affects gene expression when used as food supplement for *Drosophila*. To this end, eye colour expression in male flies of consecutive generations was used as the read-out. As controls, sibling flies of the parental generation were raised under the same conditions but without Tranylcypromine, and eye colour expression investigated in the same generations, under the same conditions, and in parallel to the treated animals. Increasing concentrations of Tranylcypromine (from 1.25 mg up to 4 mg per culture vessel) were fed to the parental fly generation (P/F0) and their F1 progenies, and eye colour analysed by optical measurement of pigment extracts. We found a significant increase in eye pigmentation in generation F1 (Student’s T test: p**) (Fig. 2a). To investigate *white* gene expression, flies were fed with 1.25 mg Tranylcypromine in water, and RNA of male heads extracted. In generation F3, a significant decrease in relative *white* gene expression compared to the control was found (Student’s T test: p**) (Fig. 2b). All data are normally distributed. However, the discrepancy found in generation F1 between eye colour (Fig. 2a) and expression of *white* (Fig. 2b) could be explained most likely by the large variability in eye colour in conjunction with the sample size since more biological replicates were used for eye colour measurements than for qRT-PCR. To summarize, Tranylcypromine feeding increased eye colour expression in treated flies of generation F1 but significantly decreased *white* gene expression in generation F3. An increase of Tranylcypromine administration beyond that is not feasible due to harmful side-effects. We observed that larval and pupal development was compromised resulting in a severe decrease in hatching rate. When using 2.5 mg Tranylcypromine as additive we observed a reduction of hatched flies to 67% to those of the control (four-fold experiment).

### Transgenerational effect of Tranylcypromine and DMSO feeding on eye colour expression

**Fig. 3:**
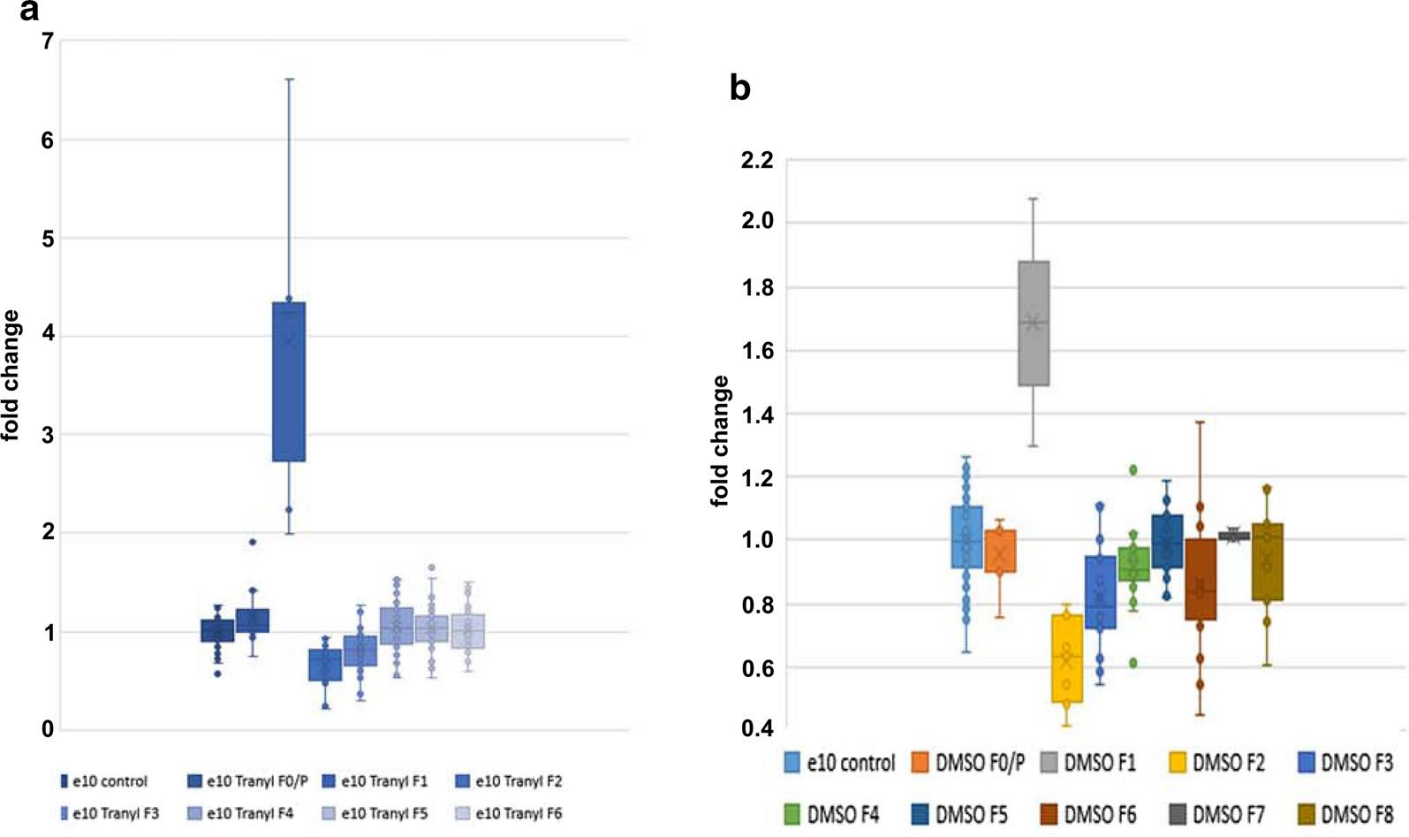
Feeding additives Tranylcypromine or DMSO affect eye colour expression. **a** Transgenerational effect on eye colour expression after feeding with Tranylcypromine in F0/P//F1 generation. For each culture vessel, Tranylcypromine was dissolved in DMSO (2.5 mg Tranylcypromine in 250 µl DMSO), diluted 1:8 in water and finally mixed with dry yeast. Eye colours of every generation were measured by using 3 × 20 male heads, i.e. a total of 60 male heads per measurement. Four individual experiments with a total of 8 biological replicates for the control and 12 biological replicates for Tranylcypromine feeding comprising a total of n individual measurements (control: n = 85 × 20 male heads, F0/P: n = 12 × 20 heads, F1: n = 6 × 20 heads, F2: n = 16 × 20 heads, F3: n = 42 × 20 heads, F4: n = 36 × 20 heads, F5: n = 39 × 20 heads, F6 n = 39 × 20 heads). Student’s T test (one-sided, homoscedastic) to control: F0/P **, F1 ***, F2 ***, F3 ***, F4 Ø, F5 Ø, F6 Ø. B) Effect of the solvent DMSO on eye colour. 250 µl DMSO was diluted 1:8 in water and the solution used for preparation of the yeast feeding mixture per culture vessel. DMSO treatment exclusively in F0/P//F1 generation. Three individual experiments comprising 8 biological replicates for the control and 5–6 biological replicates for DMSO treatment. Due to a strong effect of DMSO on viability survival of F1 flies is severely impaired. n individual measurements: control n = 92 × 20 male heads, F0/P n = 8 (× 20 male heads), F1 n = 2, F2 n = 9, F3 n = 18, F4 n = 18, F5 n = 17, F6 n = 18, F7 n = 3, F8 n = 9. Student’s T test (one-sided, homoscedastic) to control: F0/P Ø, F1 ***, F2 ***, F3 ***, F4 **, F5 Ø, F6 ***, F7 Ø, F8 Ø

In the following feeding experiments, we used 2.5 mg Tranylcypromine per culture vessel but used DMSO as solvent to increase its bio-availability. Flies were fed once in the parental (P/F0) generation and their F1 progenies, and eye colour expression measured in males of the parental and consecutive generations. We found a significant shift of eye colour expression related to the control throughout generations. The strongest effects were observed in the F1 generation, which showed an approximately fourfold increase in eye colour (p***; Student’s T test, one-sided, homoscedastic) related to the control, and a strong decrease in F2 (p***) and F3 (p***). In the succeeding generations F4, F5 and F6 no significant differences to the control were found (Fig. 3a). The increase in eye pigmentation in generation F1 of Tranylcypromine-fed male flies is exemplarily demonstrated in Additional File 1: Fig. S1. Furthermore, no obvious differences in body size or eye size between control and treated male flies were observed.

By feeding of DMSO solely, we observed similar changes in eye colour expression as in the presence of Tranylcypromine (Fig. 3b). According to Student’s T test (one-sided, homoscedastic) we found significant differences to the eye colour of control flies for F1 (p***), F2 (p***), F3 (p***), F4 (p**), and F6 ***, but not for F0/P, F5, F7 and F8. The data indicated that DMSO itself intensified eye colour expression in F1 followed by its suppression in F2 and F3, and a slow-increase towards the original level in consecutive generations. Additionally, we observed a harmful effect of DMSO itself on viability causing strong reduction of hatching in F1.

**Fig. 4:**
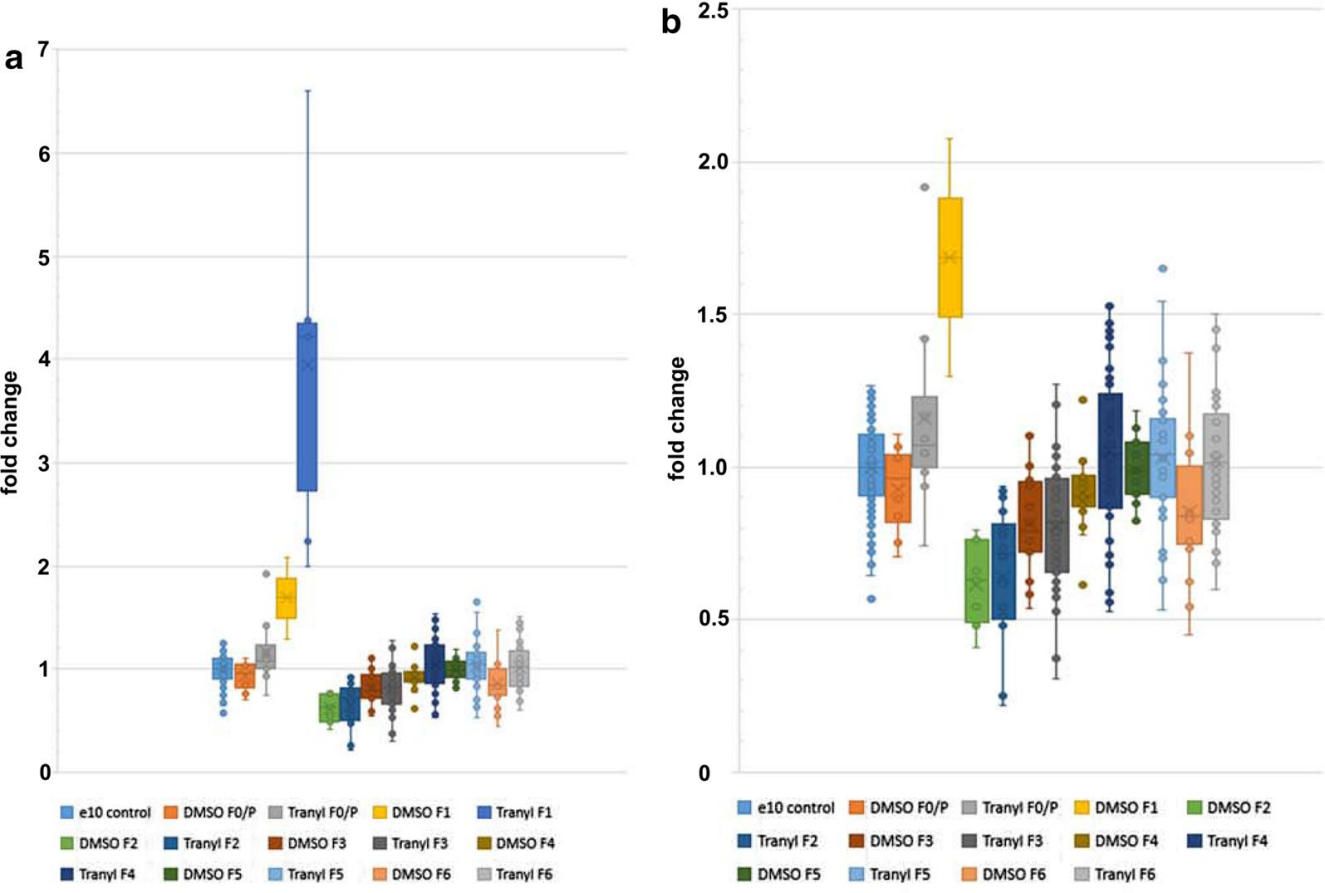
Tranylcypromine increases eye colour variation. Side-by-side comparison of eye colour changes of adult male flies treated in F0/P//F1 generation with DMSO (Fig. 3b) or Tranylcypromine dissolved in DMSO (Fig. 3a), respectively. F1 generation of Tranylcypromine/DMSO feeding included (**a**), or excluded (**b**). Student’s T test (one-sided, homoscedastic) between DMSO treatment and treatment with Tranylcypromine in DMSO in the defined generation: F0/P *, F1 Ø (p = 0.0624), F2 Ø, F3 Ø, F4 *, F5 Ø, F6 ** (p = 0.0052). Compared to the control, DMSO fed flies have a significant reduced eye colour in F4 (**) and F6 (***) whereas eye colour of F4 to F6 flies fed with Tranylcypromine/DMS0 are not significantly different to the eye colour of control flies (see Figs. 4, 6). n measurements: control n = 177 (92 + 85), all others as in the legends of Fig. 3

Side-by-side presentation of all data revealed subtle differences (Fig. 4). In the presence of Tranylcypromine, eye colour expression in F1 generation is more intense, resulting in an approximately fourfold increase compared to that of control flies, whereas DMSO itself resulted in a less than twofold increase. However, Student’s T test revealed no significant difference between DMSO and Tranylcypromine in DMSO fed flies of generation F1 (p = 0.0624), most likely due to the large variation and the small number of male flies that could be investigated because of the reduced hatching rate. Furthermore, no significant differences in eye colours between DMSO fed flies and flies fed with Tranylcypromine dissolved in DMSO (all fed in F0/P//F1 generations) were found in the consecutive generations F2 and F3, but they are significantly different in F4 (p*) and F6 (p**). Relating to the control, DMSO fed flies have significantly reduced eye colours in F4 (**) and F6 (***) (Fig. 3b) whereas no significant differences were found in F4 to F6 flies fed with Tranylcypromine in DMS0 (see Fig. 3a). Return of eye pigment expression to that of the control level in Tranylcypromine fed flies of generation F4 and F6, compared to the reduced eye pigment expression in DMSO fed flies, indicates that previous feeding with Tranylcypromine still has a supporting effect on eye pigment expression. Besides, flies fed with Tranylcypromine dissolved in DMSO showed enlarged eye colour variation throughout generations compared to flies fed with DMSO alone (Fig. 4). Thus, presumably, the supporting effect of Tranylcypromine on eye pigment expression is counteracted by a repressive effect of DMSO. The strong increase of eye pigmentation in DMSO-treated F1 flies is probably caused by its function as a polar aprotic solvent able to easily penetrate biological membranes and promoting the cellular import of other substances, e.g. of eye pigment. To conclude, Tranylcypromine and its solvent DMSO significantly affected eye colour expression at least up to generation F3, which is the first generation that has never been in contact with the additive.

### Eye pigment expression corresponds to white gene expression

**Fig. 5:**
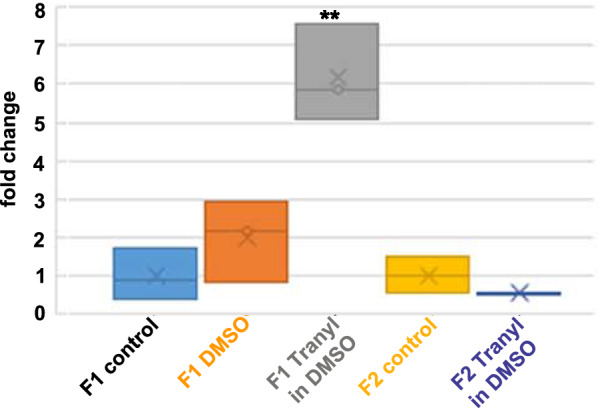
Inhibition of LSD by feeding of Tranylcypromine dissolved in DMSO in the parental generation affects H3K4 di-and trimethylation. Immunoblotting of embryonal proteins revealed an approximate sixfold increase of the relative amount of H3K4me2/3 in the F1 generation to that of control embryos (Student’s T test, p**), whereas DMSO feeding on its own did not significantly affect H3K4me2/3 level. In F2 embryos, the relative amount of H3K4me2/3 is slightly but non-significantly reduced to that of control F2 embryos but is significantly reduced compared to that of F1 embryos fed with Tranylcypromine dissolved in DMSO (p**). F1 control n = 3, F1 DMSO n = 3, F1 Tranyl in DMSO n = 3, F2 control n = 2, F2 Tranyl in DMSO n = 2

**Fig. 6:**
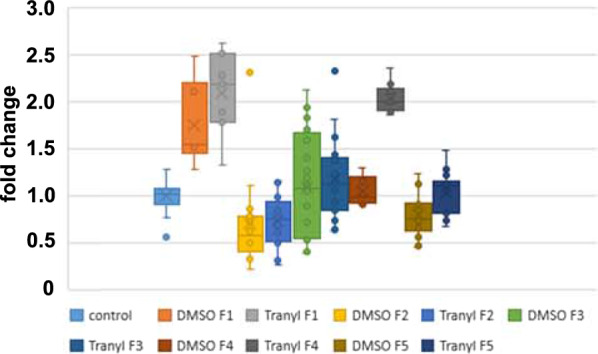
Relative *white* gene expression in consecutive generations after feeding additives. *White* gene expression was quantified using male heads from flies that were fed with either DMSO (DMSO) or Tranylcypromine dissolved in DMSO (Tranyl) in P/F0//F1 generations. Control flies raised on standard food without Tranylcypromine or DMSO. All flies were from the experiment shown in Fig. 3. Data were generated using n technical replicates from x different biological replicates (br): control br x = 7, n = 43; DMSO F1 x = 1, n = 6; T in DMSO F1 x = 1, n = 7; DMSO F2 x = 3, n = 16; T in DMSO F2 x = 3, n = 12; DMSO F3 x = 3, n = 20; T in DMSO F3 x = 4, n = 30; DMSO F4 x = 3, n = 10; T in DMSO F4 x = 4, n = 12; DMSO F5 x = 3, n = 24; T in DMSO F5 x = 4, n = 23. Student’s T test (one-sided, homoscedastic) to control: DMSO F1, T in DMSO F1, DMSO F2, T in DMSO F2, T in DMSO F4, DMSO F5 all p***, T in DMSO F3 p**. DMSO F5 to T in DMSO F5 p***

Optical measurement of eye pigment extracts revealed significant changes in eye colour expression. As expected, gene activation most likely is caused by the rise of H3K4 methylation provoked by Tranylcypromine-mediated LSD1-inhibition. In fact, H3K4 di-and trimethylation increased approximately sixfold in F1 embryos when flies were fed with Tranylcypromine dissolved in DMSO in the parental generation (Student’s T test, p**) but not when DMSO solely was fed (Fig. 5). In F2 embryos the relative amount of H3K4me2/3 is slightly but non-significantly reduced compared to that of control F2 embryos but significantly reduced compared to F1 embryos (Student’s T test, p**). The increase in H3K4 methylation indicates activation of the *white* gene, which we confirmed by quantitative RT-PCR. To this end, the relative expression of the *white* gene to *actin* as the housekeeping gene was determined and calculated as fold change to the relative expression in the controls (Fig. 6). Throughout generations, eye pigment expression obtained by optical measurements, and relative *white* gene expression are nearly congruent (see Fig. 4 and Fig. 6). *White* gene expression increased in F1, with a stronger effect when flies were fed with Tranylcypromine dissolved in DMSO than DMSO alone, decreased in F2 and slowly increased again in consecutive generations. In F4 and F5 we observed a stronger relative *white* gene expression in Tranylcypromine fed flies (fed in F0/P//F1) than in DMSO fed flies—once again reflecting eye pigmentation. All data are normally distributed. Student’s T test revealed significant differences between the control and treated flies in the following generations: DMSO F1, T in DMSO F1, DMSO F2, T in DMSO F2, T in DMSO F4, DMSO F5 all p***, T in DMSO F3 p**, and between DMSO F5 and T in DMSO F5 p***. However, there is a sole discrepancy between eye colour and *white* gene expression in generation F3 owing most likely to their large variation and the limited number of probes available for qRT-PCR. qRT-PCR was performed on individual biological replicates of one single experiment, using seven to eight technical replicates each, whereas the eye colour measurements (Figs. 3, 4) resulted from 4 individual experiments with up to 12 biological replicates. Additionally, biological replicates #5 and #6, which have reduced eye colour expression, could not be investigated by qRT-PCR due to probe limitations (Additional File 2: Fig. S2). Comparison of eye colour and *white* gene expression of individual biological replicates in F3 revealed similar tendencies (Additional File 2: Fig. S2) indicating that when all replicates, including #5 and #6, were combined an overall reduced *white* gene expression would result. An overall reduction of eye pigment expression in F3 is in all probability, by taking into consideration the large number of biological and technical replicates used for eye pigment measurement compared to the limited number of probes available for qRT-PCR, and additionally the reduced *white* gene expression in F3 of flies fed previously with Tranylcypromine in water.

## Discussion

The impact of food compounds on chromatin modifications and gene expression, and its putative inheritance was investigated using the *Drosophila melanogaster* In(1)w^*m4*^ strain. Here, the euchromatic *white* gene is transposed into the vicinity of the pericentric heterochromatin. Spreading of the inactivating effect of the pericentric heterochromatin into the euchromatic *white* gene caused stochastic inactivation resulting in a mottled eye phenotype. Expression of *white* is not imprinted and is genetically stable [49, 50]. The mottled eye phenotype is outstandingly suitable for a systems biology approach to investigate the impact of environmental conditions on gene expression by looking at one specific and easily accessible gene that variegates in expression and is environmentally susceptible.

Gene silencing and heterochromatin spreading in PEV are regulated by antagonistic and mutually exclusive histone modifications [51]. Silenced heterochromatin is characterised by methylation of histone H3 at lysine 9 (H3K9me2/3) and its reader, the heterochromatin protein 1, HP1, whereas di- and trimethylation of histone H3 at lysine 4 (H3K4me2/3) specifies genetically active euchromatin. Demethylation of H3K4 is required for subsequent di- and tri-methylation of H3K9 by SU(VAR)3–9, which is enriched in heterochromatic sites, eventually defining heterochromatin-euchromatin boundaries [52–55]. The lysine-specific demethylase 1 (LSD1) homolog of *Drosophila melanogaster,* SU(VAR)3–3 (also known as dLSD1), specifically demethylates histone H3K4me1 and H3K4me2 but not H3K9me1 or me2 [52, 56]. Spreading of heterochromatin into euchromatic regions is inhibited in SU(VAR)3–3 null cells due to elimination of H3K9 methylation in the euchromatin flanking the breakpoint [52]. *Drosophila* LSD1 (SU(VAR)3–3) is hence essential for heterochromatin formation and its mutation has a strong dominant PEV suppressor effect [8, 52]. Chemical inhibition of dLSD1, therefore, was expected to restrict heterochromatin spreading thus enabling expression of the neighbouring *white* gene as already reported for environmental factors [39, 57]. We used Tranylcypromine, an irreversible inhibitor of LSD1/2 that is widely used in clinical applications to treat depression [58]. We found that Tranylcypromine inhibits histone H3K4 demethylation in vitro in S2 cells as well as in *Drosophila* embryos. Feeding of Tranylcypromine compromised larval and pupal development and affected eye pigment expression when dissolved in water. However, its bio-availability is increased by DMSO as solvent causing a strong and long-lasting effect on eye pigmentation and *white* gene expression throughout generations. The steady presence of the LSD1 inhibitor and DMSO during embryogenesis and larval development suppressed PEV in hatched flies resulting in a strong increase in eye pigmentation. However, in the consecutive generations F2 and F3 silencing of *white* is reinforced although additives are no longer present. We observed large variation in eye pigment expression throughout subsequent generations especially when previously fed in F0/P//F1 with Tranylcypromine in DMSO. Eventually, eye pigment expression returned towards the original level, which is defined by the controls, arguing against mutations as causative for gene expression shift. However, significant changes in eye colour and *white* gene expression were observed in generations that have never been in contact with the additives, e.g. generation F3. Thus, our results demonstrate that drug-mediated alterations in gene expression are detectable also in consecutive generations that have never been in contact with the additive and that is, most likely, transgenerational epigenetic inheritance.

The inhibition of dLSD1 by Tranylcypromine presumably not only inhibited demethylation of H3K4 but also prevented methylation of H3K9 resulting in desilencing of the *white* gene in the F1 generation, as observed. Inhibition of dLSD1 in the F1 generation not only affected the establishment of chromatin states in early embryogenesis but also its propagation during development due to the permanent presence of Tranylcypromine [20, 52, 60]. Silencing of *white* is reinforced in the F2 generations, despite development from germ cells generated in the presence of the LSD1 inhibitor Tranylcypromine assuming a desilenced chromatin state. Embryogenesis and development, however, happened in the absence of the LSD1-inhibitor Tranylcypromine thus preventing the propagation of the desilenced state. Spreading of heterochromatin and reinforced suppression of *white* is most likely caused by reinforced heterochromatin signatures that are putatively fuelled by an imbalanced expression of chromatin modifiers due to previous inhibition of dLSD1. SU(VAR)3–9 plays an essential role in the establishment and maintaining of heterochromatin. The presence of parental methylated H3K9, epigenetically inherited, suffices to recruit SU(VAR)3–9, activating its methyltransferase, and in turn catalyses further H3K9 methylation to propagate heterochromatin domains [61]. Inhibition of dLSD1 acts on the euchromatin, preventing heterochromatin spreading over the euchromatin-heterochromatin boundary but did not affect the constitutive heterochromatin that preserves its repressive histone marks. These repressive histone marks later on, in the absence of the LSD1 inhibitor, may act as seeds for the propagation of heterochromatin and its spreading into the euchromatic *white* gene. Reestablishment of the euchromatin-heterochromatin boundaries and heterochromatin propagation in progenies generated in the absence of the inhibitor may account for the obvious variability of eye pigment expression. Furthermore, this variability indicated an unstable—or metastable—epigenetic state and a memory capacity of previous epigenomes [59].

The effect of DMSO itself on eye pigment expression might partially be caused by its property as a membrane-permeable solvent. DMSO is widely used in pharmacological applications as a carrier that enables the cellular import of desired substances. DMSO might thus directly affect the transport of eye pigments to increase eye pigmentation in generation F1. Additionally, DMSO is a histone deacetylase (HDCA) inhibitor directly affecting gene activity as observed for the HDCA inhibitor butyrate [57, 62]. Thus, DMSO itself seems to affect histone modifications, as observed in the increase of H3K4 di- and tri-methylation in S2 cells and F1 embryos (Figs. 1, 5) and may perturb the balanced expression of chromatin modifiers likewise as Tranylcypromine. Although we observed an effect of Tranylcypromine itself on H3K4-methylation in S2 cells and on eye colour expression in F1-flies a much stronger effect was observed when DMSO was used as solvent. It is therefore hard to discriminate the contribution of Tranylcypromine from that of DMSO. The LSD1 mutant might be helpful to identify any further effects on gene expression, whether caused by Tranylcypromine or DMSO feeding [8]. However, the transgenerational effect of DMSO is highly relevant concerning its common use in medical applications. As for LSD1, which is an oncogenic determinant, pharmacological intervention is intended but whether inter- or transgenerational side effects may occur have not been investigated so far [63].

## Conclusions

Environmental conditions and diet can cause epimutations to eventually affect gene activity in progeny as demonstrated by dietary supplementation in pregnant mammals [33, 64, 65]. Tranylcypromine inhibits demethylation of histone H3 at lysine 4, which is essential for the establishment of the euchromatin-heterochromatin boundary, and spreading of the silencing effect of the pericentric heterochromatin into the transposed euchromatic *white* gene in *Drosophila*. Feeding of Tranylcypromine and its solvent DMSO affects eye colour expression not only in treated animals but has a long-lasting effect observable also in generations that have never been in contact with the additives. Our results thus indicate that environmental conditions including food compounds may influence the phenotype of progenies for generations. Accordingly, environmental conditions including the composition of food and intake of drugs might contribute to broadening non-mendelian phenotypic variability, known as plasticity [40]. Eventually, epigenetic plasticity caused by heritable transmission of environmentally induced epigenome modifications could provide long-term adaptation to changing environmental conditions and finally facilitate phenotypic evolution. However, our results also show that the shift in gene expression, induced by a single drug administration, is non-permanent implying that a sophisticated interplay between activators and inhibitors safeguards the return to the balanced gene activity.

## Methods

### Fly strains and culture.

Fly stocks were maintained on standard cornmeal-glucose-yeast medium (per litre: 10.2 g agar, 10 g soy flour, 80 g cornmeal, 18 g brewer’s yeast, 22 g treacle, 80 g Malzin, 6.3 ml propionic acid, 1.5 g Nipagin) at 25 °C with continuous light as usual. The white mottled fly strain *e10* (*w*^*m4h*^*; CyRoi/Sco*) was obtained from Gunter Reuter, Halle [49].

All experiments were performed on mass cultures. Sibling flies were equally split into Ø 4.5 cm culture vessels containing *Drosophila* standard food with or without additives. Tranylcypromine (Calbiochem, 616,431) was either dissolved in water (1.25 mg up to 4 mg in 2 ml of water per culture vessel) or first in DMSO (2.5 mg Tranylcypromine in 250 µl DMSO) followed by dilution with 1.75 ml water resulting in a DMSO concentration of 12.5%. A filter paper placed into the culture vessel was soaked with 500 µl of the Tranylcypromine solution. Dry yeast was mixed with the remaining Tranylcypromine solution and the slurry put into the fly culture vessel. As the temperature strongly affects PEV by suppressing inactivation at high temperatures, causing an increase in eye colour, all flies were raised at constant temperature of 25 °C [39, 57]. The experiment was started by feeding the parental generation (F0/P) with the additive, and their sibling flies without the additive as control. The parental generation was removed and analysed after egg-laying, and the next generation (F1) reared up to the adult stage in the same vessel. F1 flies were then transferred into new culture vessels containing usual *Drosophila* food without further additives. F1 flies were collected and analysed after egg-laying. F2 flies were again transferred into new culture vessels without further additives and analysed after egg-laying. This scheme was carried on for several generations without further supplement addition. Flies were always transferred to new culture vessels when hatched, removed approximately 8 days after egg-laying and heads of male flies collected. In short, F0/P adults were fed on standard food with or without additives. Their progenies (F1) were raised in the same vessels and on the same food up to the adult stage and hatched flies transferred to new culture vessels without additives. Therefore, not only the parental generation but also the F1 generation were fed with the additives. Accordingly, the F3 generation is the first generation that has never been in contact with food additives.

For the analyses of eye pigment expression, the heads of male flies were collected followed by extraction in 30% acidic ethanol for 24 h at 25 °C. Usually, the eye colour in each experiment was determined using duplicate or triplicate samples, each comprising 20 male heads, from the same biological replicate. The eye colour was measured at 480 nm [21, 66]. To exclude any dosage effect, only male heads were used for all experiments since an increase in eye colour in females was observed.

As controls, sibling flies, bred under identical conditions but without food additives, were used throughout generations, and preparation and eye colour extraction for controls and treated animals were performed concurrently. The optical densities (OD480) of treated lines were always normalized to the OD480 level of the control of the corresponding generation. For comparability reasons, the arithmetic mean of the optical densities of the eye colour extracts of control flies of each individual experiment (performed in triplicate) was used as the reference (set as 1) to calculate the fold-change, i.e. in every experiment and every generation, the mean eye colour of control flies of the corresponding generation was always used as the reference.

### Cultivation of S2 cells and protein analyses

Schneider S2 cells were cultivated in standard medium (Schneider’s medium (life technologies/Thermo Fisher, #21720-024) containing 10% fetal calf serum) at 25 °C. Cells were incubated either without or with 2 µM Tranylcypromine dissolved either in water or DMSO. Cells were washed in PBS and lysed in SDS-sample buffer containing 5% ß-Mercaptoethanol, sonified and boiled. Total protein lysates were analysed by SDS-PAGE and Western blotting using rabbit anti-histone H3 (Abcam, #1794, or Cell Signaling (D1H2), #4499 T) and mouse anti-histone H3K4me2/3 antibodies (Abcam, ab 6000–100), and the fluorescent labelled secondary antibodies (goat anti-rabbit-IRDye680RD; LI-COR 925-68071, lot #C80911-11, and goat anti-mouse-IRDye800CW; LI-COR 925-32210, lot #C81106-01) [67, 68]. Images were captured by Odyssey CLx Imaging System (LI-COR) and proteins quantified using the software Image Studio™ Lite (LI-COR).

### Analyses of H3K4 methylation level in embryos

Mass cultures of *Drosophila* were raised, and sibling flies split for egg-laying on apple juice plates spotted with yeast slurry either without or with Tranylcypromine. Additionally, a filter paper soaked with either water or the Tranylcypromine solution was placed onto each agar plate. 2.5 mg Tranylcypromine was dissolved in 4 ml water, 500 µl of the solution spotted onto the filter paper placed on top of the agar plate, the rest was mixed with dry yeast and the whole slurry split onto two agar plates. Tranylcypromine dissolved in DMSO was essential as described (2.5 mg dissolved in 250 µl DMSO and diluted with 1.75 ml water for one plate). Embryos were collected 18 h after the start of the experiment. Embryos were dechorionated and kept frozen at -80 °C until lysis in SDS sample buffer by boiling for 3 min and sonication for 45 s. Proteins were separated on 15% SDS-PAGE, transferred to Hybond ECL, and proteins detected as described [67, 68]. Embryos of F2 and F3 generations were obtained by raising F1 flies fed with Tranylcypromine in P/F0/F1 generation and egg-laying on apple juice agar plates without additives.

### Quantitative reverse-transcribed PCR (qRT-PCR)

Total RNA was prepared from up to 100 male heads using peqGOLD RNAPure™ (PEQLAB, Erlangen, Germany) followed by DNaseI digestion. Complete elimination of genomic DNA was verified by PCR amplification of the *white* gene using the primer pair *white 4f/white Xr* (*white 4f* ggagcggcttcgcagagctg, *white Xr* cacggccaaaagttcgcc) that encompasses an intron resulting in a shift of the PCR product compared to that of the cDNA. cDNA was generated from up to 2 µg of total RNA using Maxima™ First Strand cDNA Synthesis Kit (Fermentas) and oligo (dT)_18_ primer. The quantitative real-time PCR was performed on CFX96TM Real-Time System (Bio-Rad) with GeneCopoeia All-in-One qPCR Master Mix (QP001; GeneCopoeia, Inc., Rockville, MD, USA). The *white* cDNA was amplified using *white 4f* (ggagcggcttcgcagagctg)/*white Xr (*cacggccaaaagttcgcc) and the *actin* cDNA using *actin* primers *Dmel_Act5C_f* (gcaacgagcgtttccgctgc)/*Dmel_Act5C_r* (tgcatacggtcggcgatgcc). The relative expression was calculated by ΔΔ*C*_t_. The specificity of the amplification reaction was verified by melting curve analyses. Measurements were done in triplicates. Fold changes were calculated to the mean of the relative *white* gene expression of the controls, set as 1.

### Statistical analyses

Data were analysed and presented using Excel. The box in the boxplots represents the 25–75th percentile. The median is given as a line, the mean by a cross. The whiskers show the minimum and maximum values inside the range given by Q1 − 1.5 × interquartile range (IQR) and Q3 + 1.5xIQR. Data were analysed for normal distribution and by Student’s T test.

## Supplementary information

**Additional file 1: Fig. S1** Feeding of Tranylcypromine affects eye pigmentation in male flies. A) The variegated eye pigmentation is obvious in untreated control flies. B) Feeding of Tranylcypromine (dissolved in DMSO) caused an increase in eye pigmentation in the F1 generation. Eye colour variegation is hardly visible. Furthermore, no obvious differences in body size between untreated and Tranylcypromine-treated male flies were found.

**Additional file 2: Fig. S2** Eye colour expression in individual biological replicates (#1 to #6) of generation F3 after feeding with Tranylcypromine/DMSO, in F0/P/F1 generations. Sibling control flies raised on standard food without additives. A) Fold change of eye colour expression by optical measurements. Fold changes were calculated to the mean optical value of the control flies of the same generation. Triplicate measurements. Same experiment as used for qRT-PCR (Fig. S2B). B) Fold change of the relative white gene expression to the control. qRT-PCR of white and actin, respectively, in individual biological replicates (#1 to #4). Between 7 and 8 technical replicates. Replicates #5 and #6 were not available for qRT-PCR.

## Data Availability

The datasets generated during the current study are available from the corresponding author on reasonable request.
